# Interferon-Lambda3 (IFN-λ3) and Its Cognate Receptor Subunits in Tree Shrews (*Tupaia belangeri*): Genomic Sequence Retrieval, Molecular Identification and Expression Analysis

**DOI:** 10.1371/journal.pone.0060048

**Published:** 2013-03-28

**Authors:** Ming-Li Li, Wen-Wen Xu, Yue-Dong Gao, Yan Guo, Wen-Ju Wang, Chao Wang, Shi-You Jiang, Andrew Willden, Jing-Fei Huang, Hua-Tang Zhang

**Affiliations:** 1 Key Laboratory of Animal Models and Human Disease Mechanisms of the Chinese Academy of Sciences & Yunnan Province, Kunming Institute of Zoology, Kunming, China; 2 Chongqing Center for Biomedical Research and Equipment Development, Chongqing Academy of Science and Technology, Chongqing, China; 3 University of the Chinese Academy of Sciences, Beijing, China; 4 State Key Laboratory of Genetic Resources and Evolution, Kunming Institute of Zoology, Chinese Academy of Sciences, Kunming, Yunnan, China; 5 Editorial Department, Kunming Institute of Zoology, Chinese Academy of Sciences, Kunming, China; NIAID, United States of America

## Abstract

Type III IFNs (IFN-λs) constitute a new subfamily with antiviral activities by signaling through a unique receptor complex composed of IFN-λs receptor 1 (IFNλR1) and interleukin-10 receptor 2 (IL10R2). As tree shrews (*Tupaia belangeri*) have shown susceptiblility to several human viruses, they are a potentially important model for analyzing viral infection. However, little is known about their IFN-λs system. We used the tree shrew genome to retrieve IFN-λs and their receptor contig sequences by BLASTN and BLASTZ algorithms, and GenScan was used to scan transcripts from the putative contig sequences. RT-PCR and bioinformatic methods were then used to clone and characterize the IFN-λs system. Due to its highest identity with human IFN-λ3, we opted to define one intact IFN-λ gene, tsIFN-λ3, as well as its two receptor subunits, tsIFNλR1 and tsIL10R2. Additionally, our results showed that tsIFN-λ3 contained many features conserved in IFN-λ3 genes from other mammals, including conserved signal peptide cleavage and glycosylation sites, and several residues responsible for binding to the type III IFNR. We also found six transcript variants in the receptors: three in tsIFNλR1, wherein different extracellular regions exist in three transmembrane proteins, resulting in different affinities with IFN-λs; and three more variants in tsIL10R2, encoding one transmembrane and two soluble proteins. Based on tissue distribution in the liver, heart, brain, lung, intestine, kidney, spleen, and stomach, we found that IFN-λs receptor complex was expressed in a variety of organs although the expression level differed markedly between them. As the first study to find transcript variants in IL-10R2, our study offers novel insights that may have important implications for the role of IFN-λs in tree shrews’ susceptibility with a variety of human viruses, bolstering the arguments for using tree shrews as an animal model in the study of human viral infections.

## Introduction

In 2003, type III interferons (IFNs), also known as IFN-λs, were described as members of a new cytokine family [1], [2]. Signaling through a heterodimeric class II cytokine receptor, IFN-λR1 (IL-28RA) and IL-10R2 (IL-10RB), IFN-λs play an essential role in the induction of an antiviral state and contribute to the initiation of adaptive immune response, similar to type I IFNs [1]–[11]. Differing from single-exon type I IFNs, IFN-λs are encoded by five to six exons. IFN-λs have been characterized in mice, zebrafish, chickens, bats, pigs, and *Xenopus* and different species had various numbers of the IFN-λ gene. Unlike the ubiquitously expressed type I and II IFNR complexes, type III IFNR is expressed predominantly in epithelial cells, consistent with a more specialized role in the immediate immune response at the sites of virus entry [6]–[8], providing a mechanism that inhibits viral replication prior to the activation of other components of the immune system.

In humans, three IFN-λ genes have been identified: IFN-λ1 (IL-29), IFN-λ2 (IL-28A) and IFN-λ3 (IL-28B); meanwhile IFN-λ1 is a pseudogene in mouse [1], [2], [7], [12], [13], four functional and one pseudo- IFN-λ (IFN-λ4) genes are present in *Xenopus* [14], and two functional IFN-λ genes in bats which are conserved with other mammalian IFN-λ sequences [15]. Likewise two IFN-λ genes, IFN-λ1 and IFN-λ3, were characterized in pigs [16], [17], while chickens and zebrafish express only a single IFN-λ [18]–[21]. Though IFN-λs have been shown to exist in several mammals, there is relatively little information regarding their presence in tree shrews (*Tupaia belangeri*), a squirrel-like mammal with similarities to primitive primates.

Tree shrews have been suggested as an important animal model as they have been shown to be susceptible to several human viruses including herpes simplex, hepatitis B, and rotavirus [22]–[24], greatly enhancing their use as models to analyze viral infection mechanisms [25]–[28]. Despite this potential utility, there is still a paucity of information on many aspects of tree shrew immunology. In the present study, we identified, cloned, and characterized IFN-λ3 and its receptor in tree shrews using RT-PCR, qRT-PCR and bioinformatics. TsIFN-λ3 has high similarity with human IFN-λ3, while both subunits of IFN-λs receptor, tsIFNλR1 and tsIL10R2, have three transcript variants in tree shrews. These results provide further evidence of the tree shrews use as important animal model for viral infections.

## Results

### Tree Shrews IFN-λ3 (tsIFN-λ3) and its Cognate Receptor Subunits in the Tree Shrew Genome

IFN-λs, IFNλR1 and IL10R2 were identified in the whole tree shrew genome sequence, available on the NCBI database (BioProject Accession: PRJNA13971, June 2006), using human IFN-λs and their receptor sequences as bait. We found an intact IFN-λ gene that we defined as the tree shrew IFN-λ3 (tsIFN-λ3), in accordance with the corresponding human gene. Furthermore, two additional IFN-λ-like sequences were also identified, but we were not able to obtain these sequences experimentally–we speculate that these other sequences may not actually be real IFN-λs but rather flukes or illusions generated during genomic analysis. This hypothesis raises the possibility only a single IFN-λ exists in tree shrews, but further evidence is required before coming to any definitive conclusions. Even though the integrated sequence of IFNλR1 was not found during genome analysis, the initiative and terminator regions of the open reading frame (ORF) were sought out, providing enough information to design oligonucleotide primers to amplify full-length cDNA. To IL10R2, an ORF sequence lacking the ATG was obtained by gene prediction. The specific tree shrew IL10R2 sequence first was amplified using IL10R2_1F and IL10R2_R. Afterward, we designed a primer, primer IL10R2_2F, based on the upstream sequence. In the future, a high-quality genome is needed to accurately portray type III IFNs of tree shrews due to the low coverage (2*) genome sequence.

### Cloning of tsIFN-λ3

TsIFN-λ3 was amplified using RNA extracted from polyinosinic-polycytidylic acid (poly I:C) stimulated-tree shrew kidney (TS-K5) cells. The sequence has been deposited in GenBank under accession number JX185489. Exon-intron analysis showed that tsIFN-λ3 has a similar genomic structure with human IFN-λ3, five exons and four introns (data not shown).

**Figure 1:**
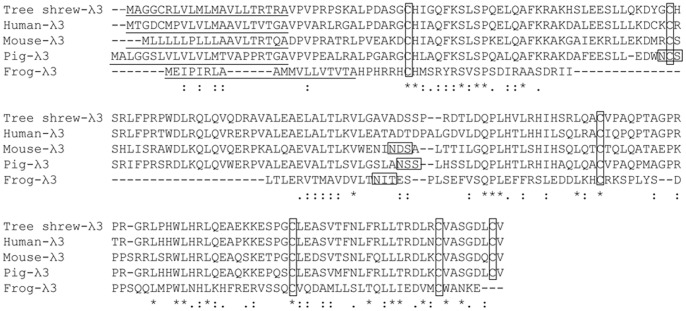
Alignment of the tsIFN-λ3 sequence with human, mouse, and frog IFN-λ3 sequences. Sequences were generated using Clustal-omega. The putative signal peptide sequences predicted by SignalP are underlined. Conserved cysteines and predicted glycosylation sites ate boxed. The identical amino acid residues are marked ‘*’, conserved and semiconserved residues are depicted by : and., respectively. Dashes indicate gaps introduced into the sequence to optimize alignment.

The deduced protein encoded by tsIFN-λ3 (shown in Fig. 1) was 194 amino acids (aa) in length and contained 21 aa signal peptides. Six conserved cysteine residues, which exist in mammals and are important for disulphide bond formation, are also conserved in tsIFN-λ3 sequence (Fig. 1).

### TsIFN-λ3 Gene is Closely Related to Human IFN-λ2 and IFN-λ3 Genes

**Figure 2:**
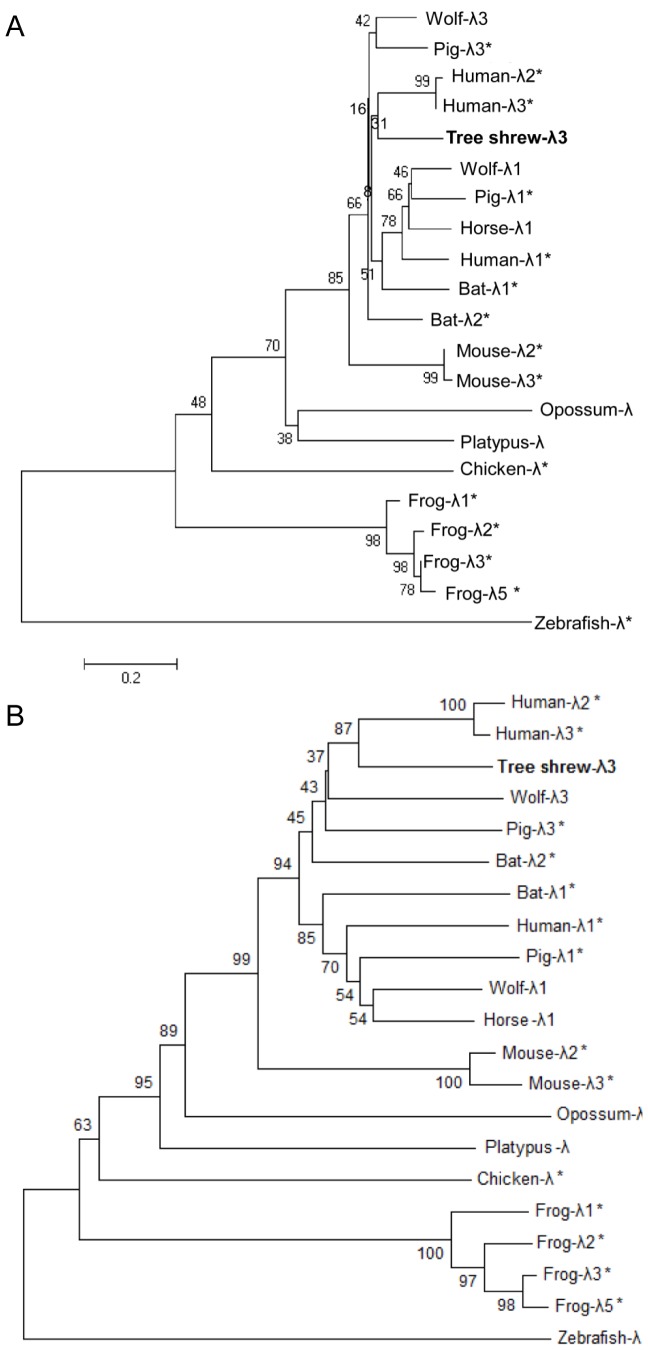
Phylogenetic analysis of representative vertebrate species based on nucleic acid and amino acid alignments of tsIFN-λ3. Unrooted phylogenetic trees were constructed using the full-length nucleic acid and amino acid sequences with the neighbor-joining method within MEGA5 and bootstrapped 10,000 times. The tsIFN-λ3 is highlighted in bold. The accession number of IFN-λ protein used in this tree can be founded in *Material and Method.* *indicate proteins that have been characterized; all other sequences are predicated based on whole-genome sequences.

To determine how the development of tsIFN-λ3 relates to other animal type III IFNs, we performed phylogenetic analysis on tsIFN-λ3 with a group of other vertebrate IFN-λ sequences based on nucleic acid alignments. Our analysis showed that tsIFN-λ3 clustered with human IFN-λ2 and IFN-λ3 (Fig. 2A). Furthermore, to determine the protein function of tsIFN-λ3 related to other animal type III IFNs, we performed phylogenetic analysis on tsIFN-λ3 with a group of other vertebrates IFN-λ sequences based on amino acid alignments, again showing that tsIFN-λ3 clustered with human IFN-λ2 and IFN-λ3 (Fig. 2B). Furthermore, the relationship between tree shrews and humans is much closer than between human and mice. Multiple sequence alignment results showed that human IFN-λ2 and IFN-λ3 share significant homology: 98% identity at the nucleotide level and 96% similarity at the protein level for the coding region, but limited homology with IFN-λ1, 71% and 74% at the protein level, respectively. In addition, multiple sequence alignment results showed eight different amino acids between human IFN-λ2 and IFN-λ3, and human IFN-λ2 is four amino acids longer than human IFN-λ3. The length of tsIFN-λ3 is the same as human IFN-λ3, and three of the eight different amino acids are identical with human IFN-λ3 amino acids. However, only one of eight different amino acids is the same as human IFN-λ2 relevant amino acid (data not shown), leading us to conclude that the IFN-λ retrieved by us in this experiment is tsIFN-λ3 and not tsIFN-λ2. The phylogenetic tree we constructed based on nucleic acid and amino acid showed that type III IFNs can be separated into two branches: with IFN-λ2 and IFN-λ3 clustering in one branch, and IFN-λ1 existing as an individual branch.

We used 14 reported IFN-λ sequences to analyze the identity in different species and among the same species (Table 1). Identities of IFN-λs from different species range between 20% (tsIFN-λ3/fIFN-λ3) and 77% (tsIFN-λ3/hIFN-λ3) for ORF amino acids sequences. As for intraspecies identities, interestingly they range from 66% (pIFN-λ1/pIFN-λ3) to 96% (hIFN-λ2/hIFN-λ3). Furthermore, these intraspecies identities are more strongly related than identities between type I and type III IFNs (data not shown).

**Table 1:** Similarity of ORF sequences between reported functional IFN-λ subtypes.

	tsIFN-λ3	hIFN-λ1	hIFN-λ2	hIFN-λ3	mIFN-λ2	mIFN-λ3	pIFN-λ1	pIFN-λ3	bIFN-λ1	bIFN-λ2	cIFN-λ	fIFN-λ1	fIFN-λ2	fIFN-λ3	fIFN-λ5
tsIFN-λ3	/														
hIFN-λ1	69	/													
hIFN-λ2	76	**71**	/												
hIFN-λ3	77	**74**	**96**	/											
mIFN-λ2	64	62	66	63	/										
mIFN-λ3	61	58	62	67	**93**	/									
pIFN-λ1	66	74	63	65	56	54	/								
pIFN-λ3	72	67	72	74	66	62	**66**	/							
bIFN-λ1	62	68	64	65	59	56	63	62	/						
bIFN-λ2	72	70	67	72	62	58	73	75	**69**	/					
cIFN-λ	37	34	38	37	33	31	38	33	34	38	/				
fIFN-λ1	27	25	26	27	28	27	26	28	26	29	28	/			
fIFN-λ2	27	23	25	25	26	25	25	28	27	29	31	**87**	/		
fIFN-λ3	20	21	22	22	24	22	21	23	28	25	32	**69**	**75**	/	
fIFN-λ5	25	22	25	25	25	25	25	24	24	26	29	**85**	**89**	**78**	/

hIFN: human IFN; mIFN: mouse IFN; pIFN: pig IFN; bIFN: bat IFN; cIFN: chicken; fIFN: frog IFN. Values are given as percentages and calculated from the multiple alignment of ORF sequences. Level of similarity was calculated for each pair of aligned sequences as the number of identical resudues divided by the number of aligned residues, excluding positions with gaps and flanking unaligned posittions in both sequences. The bold numbers indicates intraspeices similarities.

### Both tsIFNλR1 and tsIL10R2 have three Transcript Variants

Full-length sequences of the acquired tree shrew IFNλR1 (tsIFNλR1) and IL10R2 (tsIL10R2) were amplified using RNA extracted from TS-K5 cells. The nucleotide sequences have been deposited in GenBank under accession numbers JX402614-16 and JX402617-19, respectively.

**Figure 3:**
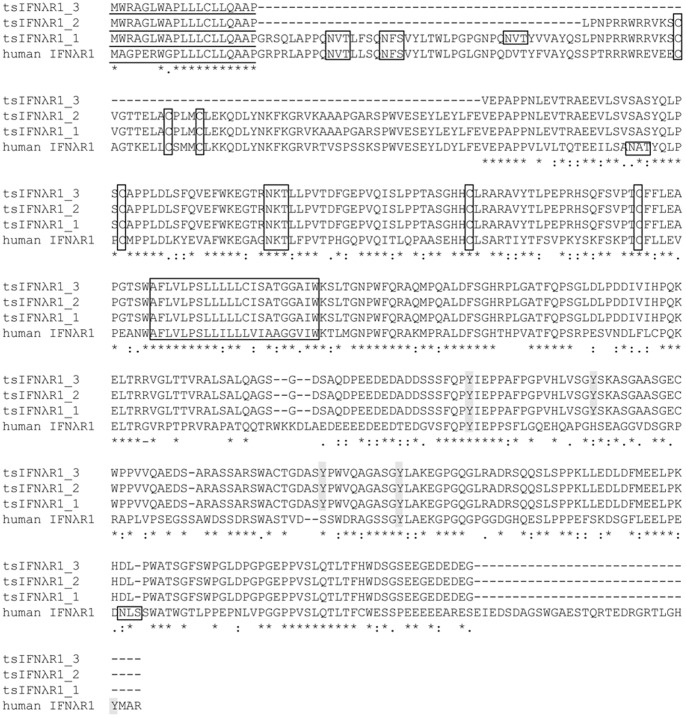
Alignment of tree shrew and human IFNλR1 sequences. Analysis was generated using Clustal-omega. Putative signal peptide sequences predicted by SignalP are underlined. Identical amino acid residues are marked ‘*’, conserved and semi-conserved residues are respectively depicted by : and. Dashes indicate gaps introduced into the sequence to optimize the alignment. Tyrosine residues are shadowed and predicted glycosylation sites, conserved cysteine and transmembrane regions are boxed.

In tree shrews, we found 3 transcript variants of tsIFNλR1–tsIFNλR1_1, tsIFNλR1_2 and tsIFNλR1_3–in tree shrews. TsIFNλR1_1 represents the longest transcript, containing 1458 base pairs (bp), encoding a protein of 485aa with a 19 aa putative signal peptide (Fig. 3). A 22 aa transmembrane region (AFLVLPSLLLLLCISATGGAIW) was seen between 227–249, separating the protein into an extracellular region of 208 aa without the leader peptide and an intracellular region of 236 aa. TsIFNλR1_2 and tsIFNλR1_3 are respectively 1332bp and 1149bp in length, encoding transmembrane proteins that are 43aa and 103aa shorter than tsIFNλR1_1 in the extracellular region, respectively. Multiple sequence alignment results demonstrated that human IFNλR1 and the full-length tsIFNλR1 share significant homology: 76% identity at the nucleotide level and 62% similarity at the protein level. Likewise, there are 6 conserved cysteine residues in the extracellular region in human IFNλR1, which are conserved in tsIFNλR1_1 and tsIFNλR1_2, while only 3 of these 6 cysteine residues exist in tsIFNλR1_3. Moreover, tsIFNλR1_1 has 4 glycosylation sites, resulting in a stable protein structure, while only one site exists in tsIFNλR1_2 and tsIFNλR1_3. Both the tree shrew and human IFNλR1 share a similar sequence, though only 2 out of 3 tyrosine residues in the intracellular domain of human IFNλR1 are conserved in the orthologous tree shrew gene. Additionally, tsIFNλR1 contains 2 more tyrosine residues.

**Figure 4:**
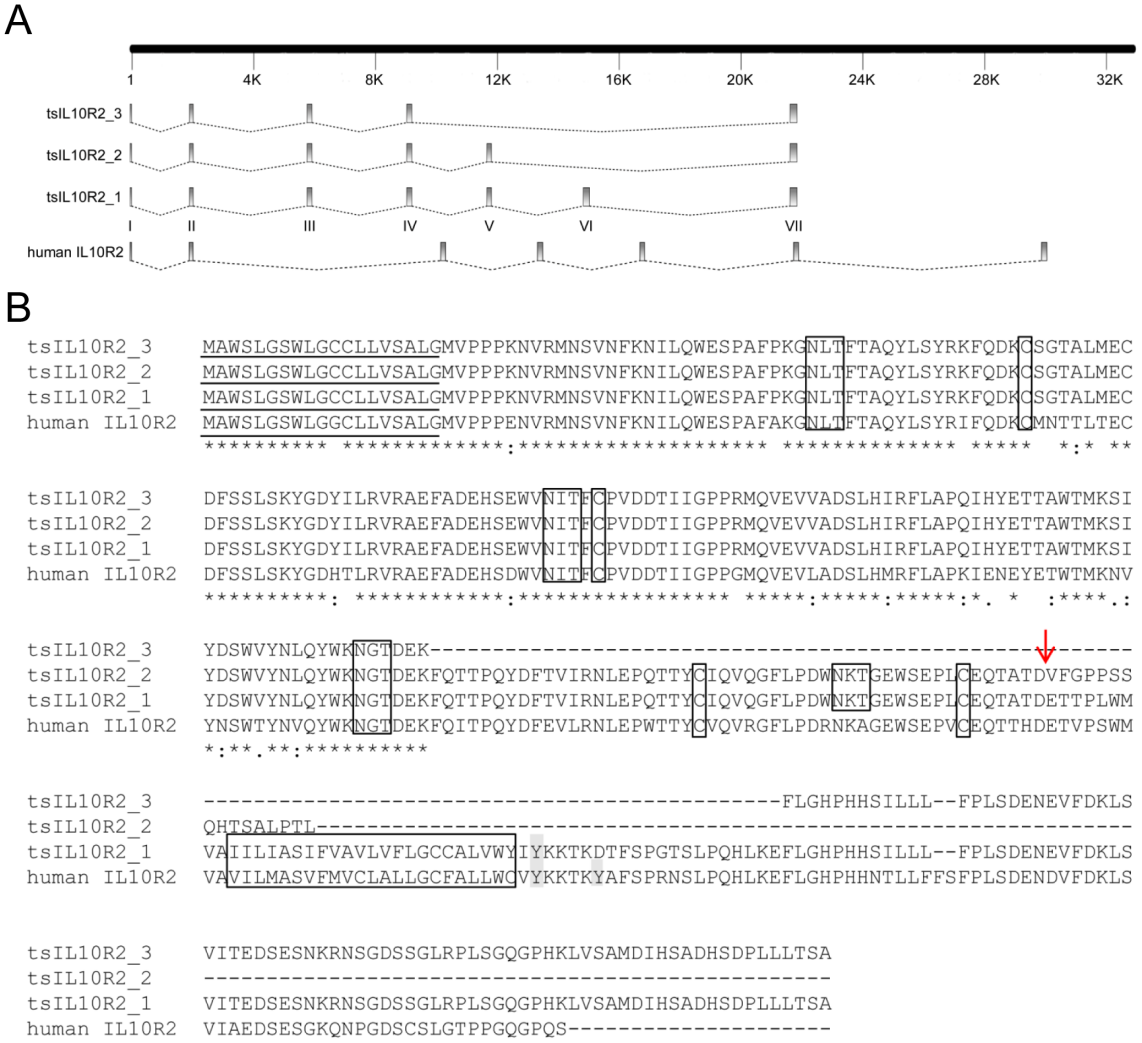
Comparison of tree shrew IL10R2 (tsIL10R2) with human IL10R2. A, Gene organizations of tsIL10R2 genes compared with that of corresponding human IL10R2 gene. Exons are drawn as rectangles and introns are shown as dotted lines. B, Alignments of the deduced amino acid sequence of tsIL10R2 genes with human IL10R2 sequences. Notes are same as in Fig. 3.

Similarly, tsIL10R2 has three transcript variants–tsIL10R2_1, tsIL10R2_2 and tsIL10R2_3. The corresponding tree shrew genomic sequence shows that tsIL10R2_1, the longest transcript variant, contains 7 exons and 6 introns in the corresponding ORF (Fig. 4A). Although 7 exons were found in human IL10R2, the second and the sixth intron of tsIL10R2 are shorter than corresponding regions in human IL10R2. TsIL10R2_2, 231 aa in length, lacks the sequence that corresponds to the transmembrane domain containing exon VI, leading to a frame shift in the exon VII-encoded sequence with an alternative, premature stop codon. As shown in Fig. 4B, the amino acid sequence changed at the end of exon V due to this frame shift. We accordingly predicted that this variant would encode a secreted receptor in which the transmembrane and intracellular sections disappeared. TsIL10R2_3 lacks the sequence corresponding to exon V and VI, causing the extracellular domain to be 58 aa shorter, the intracellular domain 21 aa shorter, and the transmembrane region disappears entirely (Fig. 4A and 4B).

Sequence alignment revealed that both human and tree shrew IL10R2 have a 19 aa putative signal peptide. TsIL10R2 shared 78% aa identity with human IL10R2 (Fig. 4B). A 23 aa transmembrane region (IILIASIFVAVLVFLGCCALVWY) was found between 224–246, separating the protein into an extracellular region of 204 aa without leader peptide and an intracellular region of 98 aa. One extra and three conserved glycosylation sites exist in the extracellular region of tsIL10R2. TsIL10R2_3 has 4 conserved cysteine residues in the extracellular region and only 1 tyrosine residue in the intracellular region that is used for recruiting signal transducer and activator of transcription (STAT). Due to the shorter-intracellular region in tsIL10R2_2 and tsIL10R2_3, the tyrosine residue disappears, resulting in no STAT recruitment region exists in these two transcript variants.

**Figure 5:**
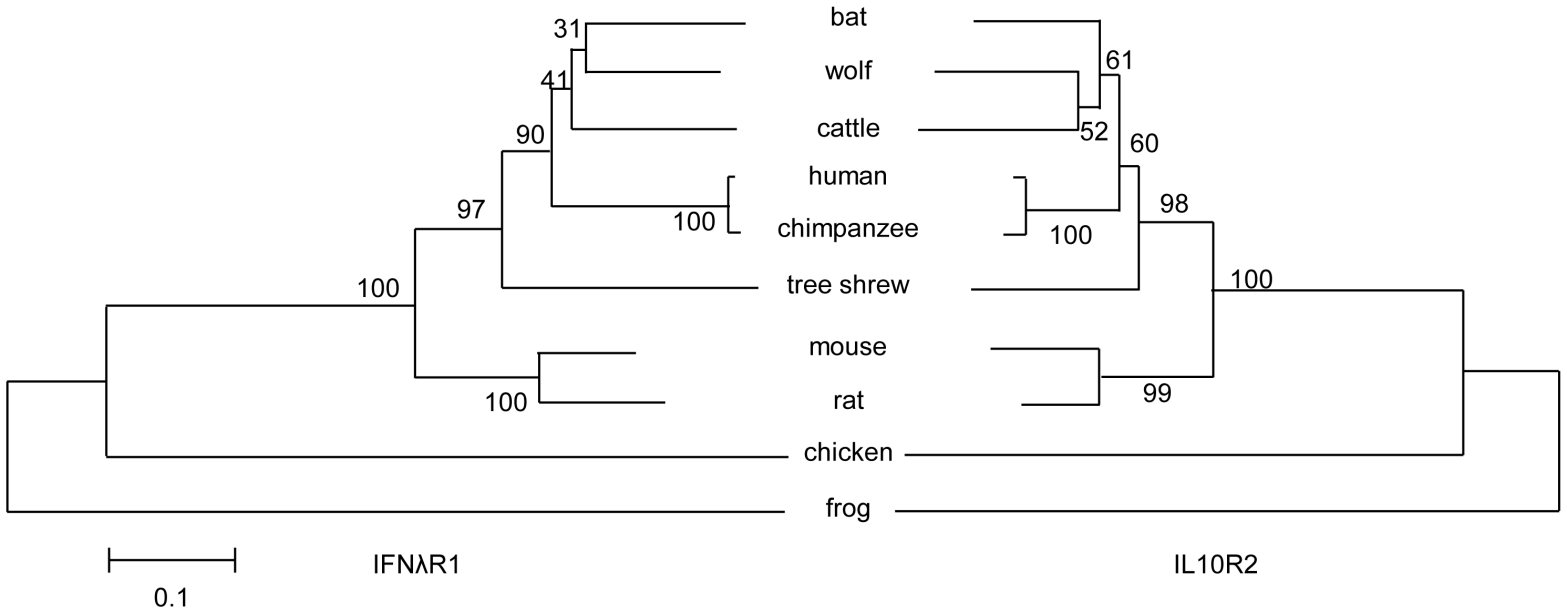
Phylogenetic analysis of tsIFNλR1 (left) and tsIL10R2 (right) within representative vertebrate species. Notes are same as Fig. 2.

Phylogenetic analysis of the tsIFNλR complex with representative vertebrate species indicated that IFNλR1 and IL10R2 had a similar evolutionary relationship (Fig. 5). Moreover, the tsIFNλR complex has higher identity with the human IFNλR complex than other widely-used animal model IFNλR complexes, such as those in both mice and rats.

### Both tsIFNλR1 and tsIL10R2 have Typical Characteristics of Class II Cytokines Receptor Family

**Figure 6:**
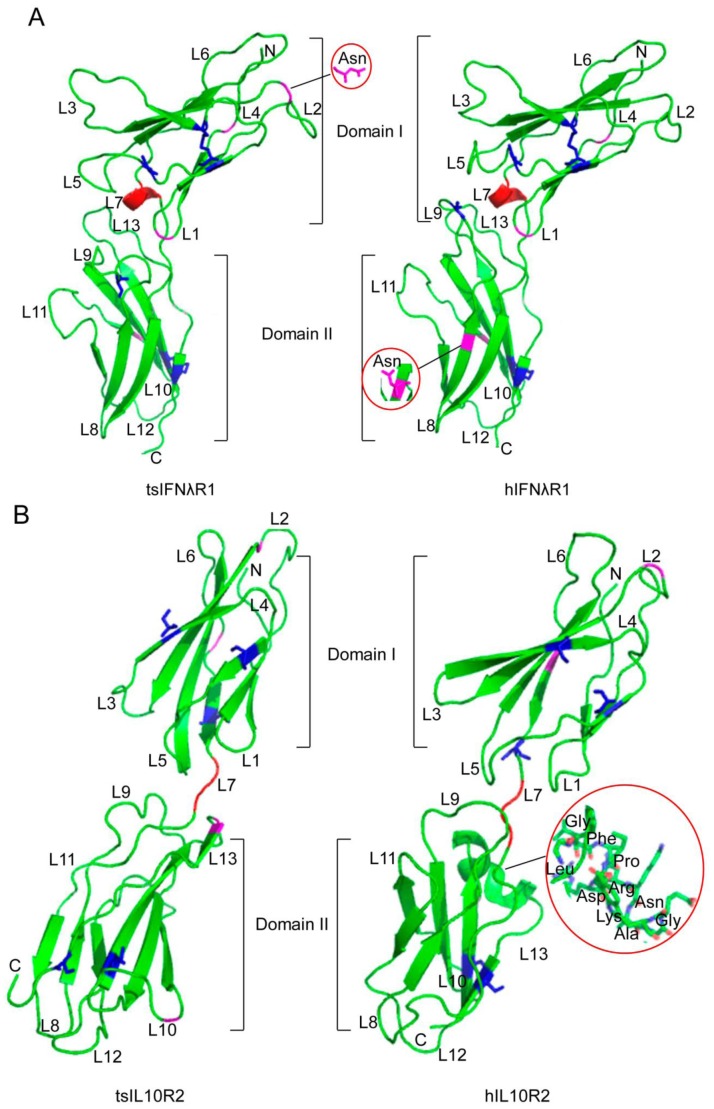
Structures of the IFNλR complex. A, Structure of tree shrew IFN-λR1 (left) compared with human IFN-λR1 (right). B, Structure of tree shrew IL10R2 (left) compared with human IL10R2 (right). Glycosylation sites are in purple (Asn), cysteines are in blue and are shown by sticks (Cys). Regions used to connect two domains are in red. Loops found in the receptor are labeled, from L1 to L13. The N and C termini are labeled as well.

To further understand the pattern of ligand-receptor interaction, we determined the crystal structure of tsIFNλR1 and tsIL10R2 (Fig. 6A and 6B). Both contain two β-sandwich domains, D1 and D2, each of which is formed by a sandwich of two β sheets (with each sheet composed of three or four stands), suggesting they have typical class II cytokine receptor family characteristics. Compared to human IFNλR1, one of the four N-glycosylation sites is different in tsIFNλR1. For IL10R2, the main difference between tree shrew and human is the portion that connects the D1 and D2 domain with an extra α-helix exists in human IL10R2. Since the function of this extra helix has not been previously reported or studied, we cannot predict any potential significance. Though differences between the tsIFNλR and human IFNλR complexes exist–in particular the N-glycosylation sites, cysteine residues, and the number of disulfide bond–the integral structures of tsIFNλR1 and tsIL10R2 are highly similar to their corresponding human proteins.

### TsIFN-λ3 mRNA is Produced Early in the Response to Poly I:C Transfection; TsIFNλR1 and tsIL10R2 are Transcribed in a Variety of Organs in Tree Shrews

**Figure 7:**
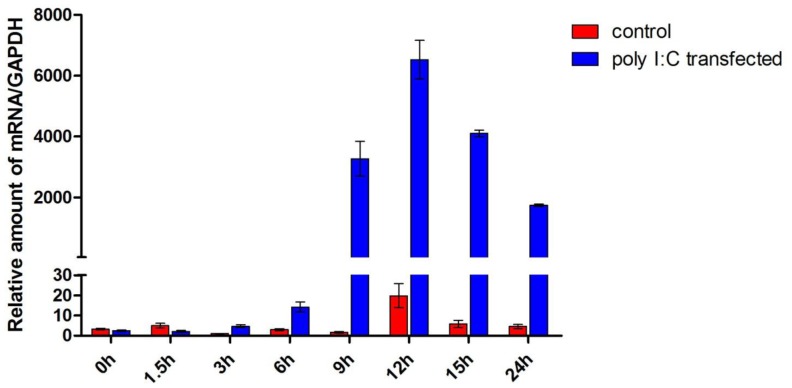
Expression patterns of tsIFN-λ3 on poly I:C transfection in TS-K5 cells. Cells were transfected with poly I:C and collected at the indicated times. tsIFN-λ3 mRNA was measured by qRT-PCR. Data were normalized against the house-keeping gene GAPDH. Data are mean values of two separate experiments, and the error bars represent SEMs.

The TS-K5 cells were transfected with poly I:C, then total cellular RNA was isolated at different times to determine the production kinetics of tsIFN-λ3. The poly I:C-mediated induction of tsIFN-λ3 was time-dependent in the TS-K5 cells. TsIFN-λ3 was induced as early as 3h, rapidly peaked at 12h, decreased by 24h (Fig. 7).

**Figure 8:**
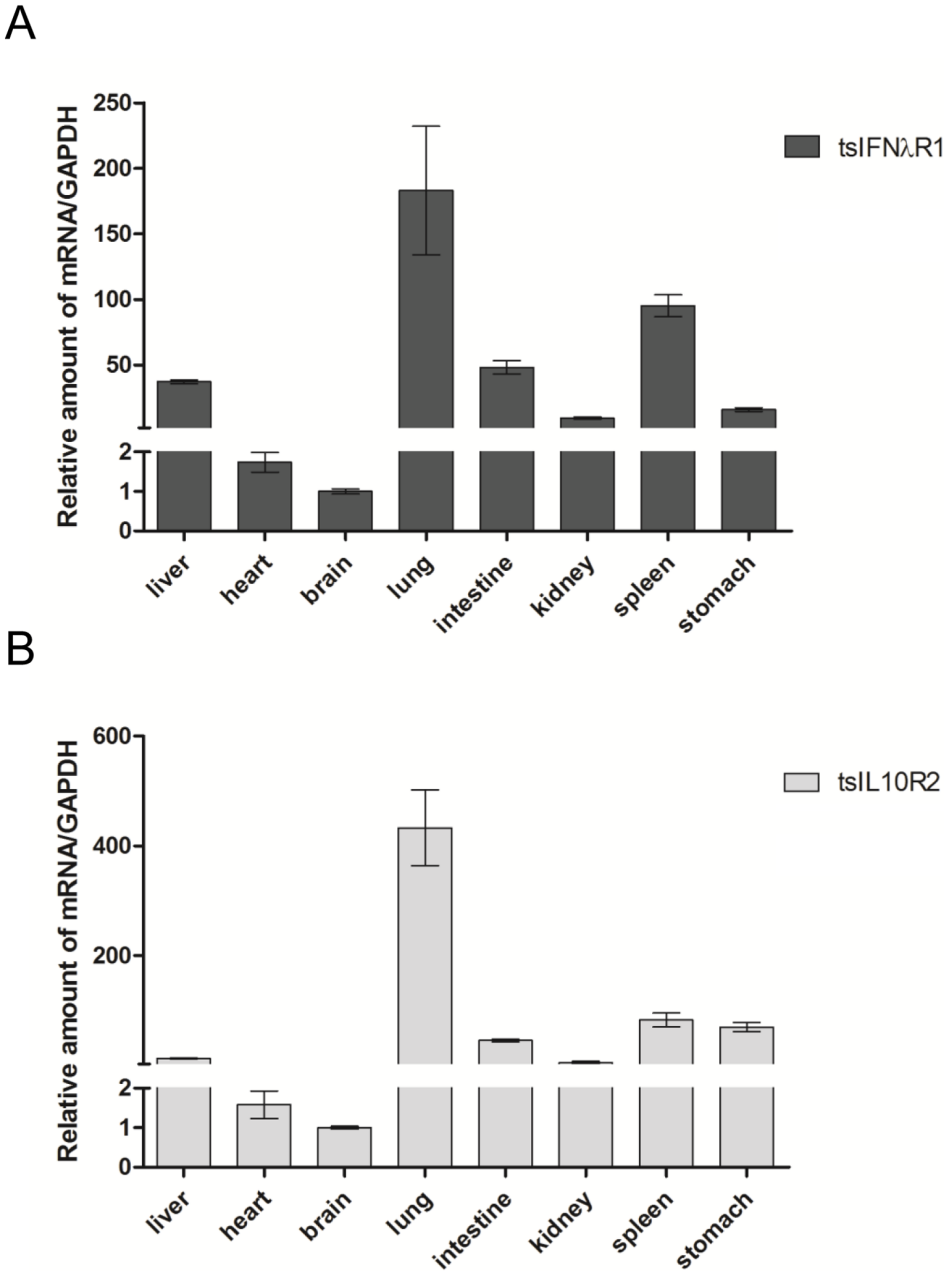
Tissue distributions of tsIFNλR1 and tsIL10R2 in healthy tree shrews. Tissues including liver, heart, brain, lung, intestine, kidney, spleen, and stomach were collected from two male tree shrews. Relative amounts of (A) tsIFNλR1 and (B) tsIL10R2 mRNA were measured by qRT-PCR and normalized against GAPDH.

In contrast to IFN-λs, which needs compulsory induction, the expression pattern of their receptor subunits is constitutive. We examined eight tree shrew organs including the liver, heart, brain, lung, intestine, kidney, spleen, and stomach to examine the tissue distribution of tsIFNλR1 and tsIL10R2. The data showed that tsIFNλR1 and tsIL10R2 are transcribed in all of the organs. Furthermore, the expression level of tsIFNλR1 and tsIL10R2 are lowest in heart and brain and different in other six tissues (Figure 8).

### TsIFN-λ3 Induces the Production of ISGs

**Figure 9:**
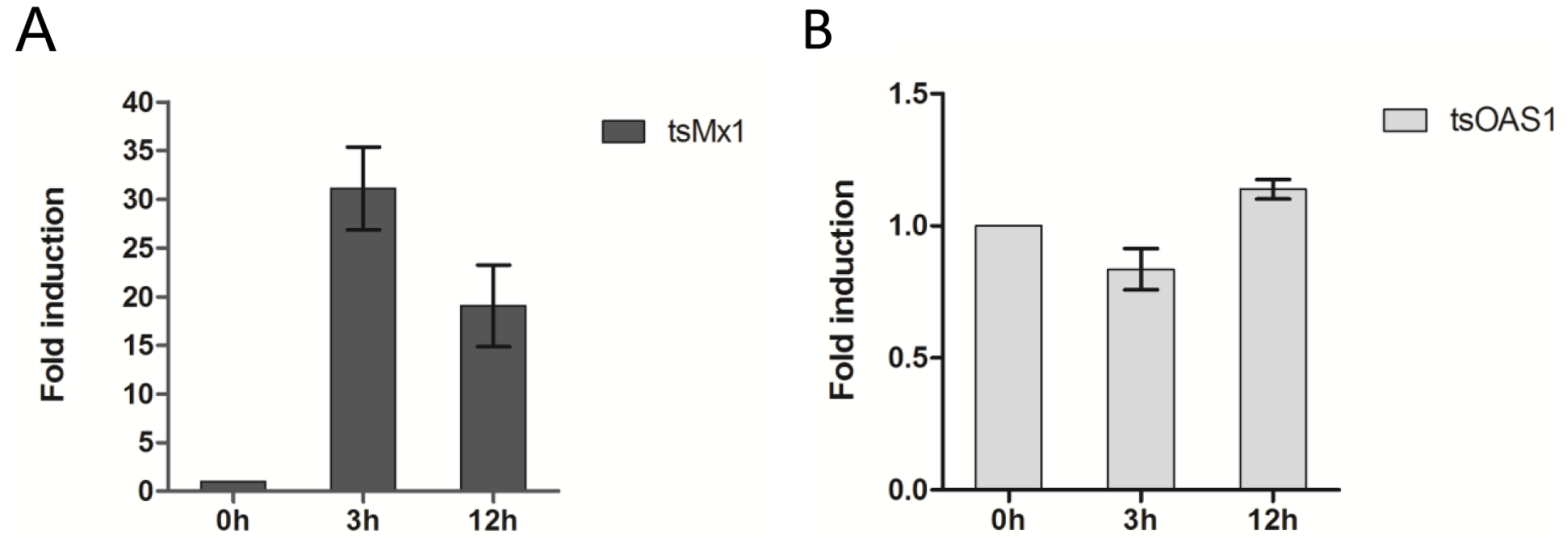
Induction of ISGs by tsIFN-λ3 in TS-K5 cells. Cells were stimulated with tsIFN-λ3 containing (or mock containing) TS-K5 cell medium for 6h. Cellular RNA was extracted and mRNA level of tsOAS1 (A) and tsMx1 (B) was measured by qRT-PCR. Data are shown as fold change of expression obtained from comparison of the tsIFN-λ3 stimulated and normal lipofectamine™ 2000-treated culture medium.

In order to test the function of tsIFN-λ3, all of the supernatant from poly I:C transfected TS-K5 cells, which expressed tsIFN-λ3 as shown in Fig. 7, was used to stimulate the TS-K5 cells. The supernatant of Lipofectamine™ 2000 treated TS-K5 cells was used as mock control. Compared to the control, tsIFN-λ3 resulted in 31-fold induction of Myxovirus resistance 1(Mx1) at 3h, while the induction of 2-prime, 5-prime-oligoadenylate synthetase 1 (OAS1) was not found (Figure 9). At 12h, about 19-fold induction of Mx1 and 1.2-fold induction of OAS1 were found.

## Discussion

Here, we first obtained the full-length ORF of tsIFN-λ3 and its corresponding receptor subunits, tsIFNλR1 and tsIL10R2, based on the current tree shrew genome using sequence alignment and gene prediction. Our experimental results showed that tsIFN-λ3 contained many conserved features compared with other spices and was produced early in response to poly I:C transfection. Both tsIFNλR1 and tsIL10R2 have three transcript variants and can express in a variety of tissues. These data represents an important step in understanding the antiviral mechanisms of tree shrews IFN-λs.

The lack of suitable in vitro infection systems and convenient animal models has greatly hampered the progress of HBV research [29]. After the development of tests for HBV surface antigen in the late 1960s, chimpanzees were the first animals found to be susceptible to HBV [30]. Although experimental and naturally occurring infections with HBV have been reported in gibbons, orangutans and rhesus monkeys, there are no reports demonstrating the development of hepatic lesions and elevation of hepatic enzymes in these animals [31]–[35]. Nonetheless, the large size, strong ethical constraints, and high costs of monkeys severely restrict their utility as models for research. In contrast to larger primates, tree shrews (*Tupaia belangeri*) are interesting mammalian group possessing characteristics of both advanced insectivores and primitive primates. Due to their small body size, short reproductive cycle and the low-cost of their maintenance, tree shrews have been suggested as alternative models to primates in biomedical research [25]–[28]. Furthermore, several papers have described the tree shrew as a promising and effective model for the ongoing study of HCV entry and replication [36]–[38]. In 2010, Amako et al. (2010) reported a longitudinal study which followed HCV-infected tree shrews over a 3-year period after inoculation with hepatitis C from a patient or viral particles from full length cDNA. Moreover, serum from infected tree shrew was harvested and inoculated into naïve tree shrew resulting in acute infection, demonstrating effective replication and potential transmission of HCV.

Nowadays, tree shrews are being studied in many aspects. A low-coverage (2*) genome of the tree shrew was used as one of 29 mammalians to identify the functional elements in the human genome [39]. The transcriptome, gene expression and a high-class genome of the tree shrew are currently being sequenced to establish tree shrew hepatocytes as a model for studying HBV infection by National Institute of Building Sciences (NIBS) of America (NCBI accession: PRJNA87013) and the Beijing Genomics Institute of China (NCBI accession: PRJNA169406). Currently, the entire transcriptome of the Chinese tree shrew is also being sequenced in our lab in order to dissect their evolutionary status. Here, based on this publicly available 2* tree shrew genome, we identified IFN-λs and corresponding receptor subunits in order to gain insight into the tree shrew IFN-λs. Nonetheless, this genome has low sequence coverage (2*), causing some artifacts and gaps. Here, for the first time we report the characterization of tsIFN-λ3 and its two receptor subunits, providing substantive evidence for IFN-λs antiviral activity analysis as well as useful methodology to identify interesting genes from genomes. To gain insight into the mechanisms responsible for the control of viral replication in this species, we will begin an investigation of the whole IFN system using high coverage genome in the near future.

Given the natural desire to utilize animal model data in a way that is both meaningful and relevant to studying humans, we have made an effort to determine a probable ortholog between the IFN-λ3 genes in humans and tree shrews. A common bioinformatics approach to doing so is comparing the similarity of related genes between different species. The IFN-λ3 genes in both human and tree shrew appear highly similar, giving another strong motivation to assign the name of tree shrew genes. This approach to ortholog assignment assumes that the ancestral species of tree shrew and human also had a high identity which was conserved for nearly 100 million years and is still present today [13], [40]. However, the phylogenetic tree we built using the IFN-λ3 nucleotide sequences from humans, mice, tree shrews, pigs, bats, etc., showed that primate-rodent speciation occurred before multiple gene loci duplicated and diverged from one another. Accordingly, the most simple and succinct interpretation is that all current IFN-λ genes arose from a single IFN-λ gene present in an ancestral species (Fig. 2). This hypothesis can easily be proven given the widely varying number of IFN-λs present among vertebrate species. Regardless, the reality that tree shrews and humans have a nearly identical IFN-λ3 requires a more recent coincidence of either gene duplication or gene conversion, as it is unlikely that the present day genes in both species are the direct product of gene duplication or conversion from a multiple gene clusters in the human-tree shrew ancestor.

In a previous study, we provided a brief description of how we identified the IFN family in the tree shrew genome and predicted crystal structure of tsIFN-λ3. Though there are some small differences in cysteine position and N-glycosylation numbers between tsIFN-λ3 and human IFN-λ3, we did find the structure of tsIFN-λ3 to be similar to human IFN-λ3 [41]. Our analysis of IFN-λs indicates that occasionally, some of the identities of among different species are higher than some of the identities among members of the same species, whereas identities of IFN-λs among the same species are all very strong. It is therefore possible that different selective pressures act on IFN-λ subtypes in a given specie; this could be related to a species specific functional specialization. Moreover, this may also explain why some of IFN-λ subtypes are biologically inactive and accordingly correspond to pseudogenes that rapidly diverged from functional IFN-λ sequences, due to the absence of selective pressure.

The evolutionary origins of both type I and III IFNs are intertwined. Only one family of IFNs has been found in present-day fish, and appears more similar to type I IFNs, while the multi-exon gene structure is identical to type III IFNs [19], [20], [42]. Based on this discovery Fox et al pointed out that type I and type III IFNs originated from a single IFN gene family [40]. However, these two types of IFNs induce cell signaling through their unique receptor complex. There is no significant sequence homology between type I and III IFNs, which cannot be explained by duplication and variation events, and no confirmed evidence as to the earlier proposed relationships. If this is the case, perhaps we can say there is no evolutionary relationship between these two family IFNs and organisms simply want to use two different families to perform identical roles to enhance the dynamic procedure.

During this study, we found that both tsIFNλR1 and tsIL10R2 have three transcript variants. We confirmed this result by three separate experiments. To date, IFNλR has been characterized in humans, mice, rats, bats, and *Xenopus* [1], [2], [7], [19], [43]. And only human IFNλR1 has different transcript variants, encoding a single soluble and two transmembrane receptors. Here, we have for the first time found different transcript variants of IL10R2 that have not been found in any other animals. Furthermore, due to the different extracellular regions of tsIFNλR1, we propose that these 3 variant forms may have a different affinity for binding to IFN-λs, thus providing a pivotal step in regulating IFN-λs signaling. Similarly, different extracellular domains exist in the 3 transcript variants of tsIL10R2, and may therefore have a different affinity for binding of IFN-λs as well. Undoubtedly, due to the lack of transmembrane and intracellular regions, tsIL10R2_2 is likely to be a fake receptor that can interact with IFN-λs but cannot trigger pathways related to immune response. Furthermore, although tsIL10R2_1 contains a partial intracellular region, the tyrosine residues used as docking sites for STAT recruitment and activation lost, resulting in tsIL10R2_1 becoming a protein, which cannot induce cell signaling after binding to its ligands. We hypothesized that these IFN-λ receptor variants evolved to mediate unique biological functions. In other words, the soluble tsIL10R2 protein may be a naturally occurring receptor antagonist that limits signaling through the membrane-bound form of this receptor.

In mounting an IFN-mediated immune response, some organisms have developed the ability to regulate IFN-mediated signal transduction. Like all cytokines, the ability of responding to IFN is completely dependent on the presence of its cognate receptor on the surface of the target cell. Accordingly, one of the major mechanisms used by the organism to regulate the strength and duration of the IFN response is done via the regulation of receptor expression levels, which thereby alter the cytokine-specific responsiveness of the target cell. Our results indicated that tsIFNλR1 and tsIL10R2 express in all of the eight tissues and the expression level are different. There are severe side-effects associated with type I IFNs therapy when used clinically for the treatment of viral infections and various cancers. The more limited tissue expression of IFN-λ receptors then suggests that IFN-λs may exhibit less toxicity than type I IFNs when administered clinically as a therapeutic antiviral agent [11], [44], [45]. In light of this finding, an important future task is establishing the tools that can determine the distribution of the functional type III IFNs receptor complex in vivo. We likewise expect a closer relationship can be found that will aid in our understanding of the specific characteristics of IFN-λs.

The IFNλR1 chain serves as a unique subunit of the type III receptor complex and is critical to ligand binding specificity [44]. Likewise, the chain has a larger intracellular domain that is associated with Jak1 tyrosine kinase, and is phosphorylated on Tyr residues after receptor engagement, which subsequently drives recruitment of various signal transducers to the receptor complex [46], [47]. On the contrary, IL10R2, which is expressed in all tissues, also forms part of the receptor complex of several members of the IL-10 cytokine family, including IL-10, IL-20, IL-22 and IL-26 [48]. Due to IL10R2 activating multiple cytokine complexes, the cellular phenotype of IL10R2 single nucleotide polymorphisms (SNPs) are associated with diseases, such as hepatitis B virus (HBV) persistence and on the like [48], [49]. For this reason, using tree shrews as a successful HBV chronic infection models requires looking for the effective SNPs of IL10R2 in future studies that may more chronic infection models viable.

Genome-wide association clinical studies have recently revealed that variations near the IFN-λ3 gene are associated with the outcome of HCV infection [3], [50]–[52]. The effects of IFN-λ3 polymorphism have been investigated for a number of aspects of response to HCV infection and treatment, including response to therapy, natural elimination of the virus, and changes in gene expression and lipid metabolism [53]. While the antiviral activity of IFN-λs against several viruses has been reported, the effect of IFN-λs on HBV infection remains obscure. We conjecture that a close relationship also exists between IFN-λ3 and HBV. From the point of view of IFN-λ3, we expect to be able to create chronic tree shrew HBV animal models by changing tsIFN-λ3.

In summary, we have identified the primary, if not all, members of the tree shrew IFN-λs family. To our knowledge, these results provide the first molecular characterization of tsIFN-λ3 and its receptor complex, thereby demonstrating that the tree shrew IFN-λs system is similar to the human IFN-λs system. The data we have provided is fundamentally useful in furthering our understanding of the tree shrews IFN system, a crucial step in utilizing tree shrew as an effective animal model of viral infection.

## Materials and Methods

### Ethics Statement

All procedures related to animal subjects were reviewed and approved by the internal review board of Kunming Institute of Zoology, Chinese Academy of Sciences.

### Animal Preparation and Isolating and Culturing of Tree Shrews Kidney-derived (TS-K5) Cells

Three 4-month-old male tree shrews were anaesthetized by 100 µl ketamine hydrochloride and then gently euthanized for dissection using methods approved by Animal Ethics Committee. Then, we removed the kidneys from the tree shrews under sterile conditions, first cut finely using a scalpel. Cold 0.25% trypsin in PBS containing 200mg/L disodium EDTA was added to the prepared tissues and placed at 4°C overnight. The tubes containing the harvested tissues were then incubated at 37°C on a shaking platform for 1 h. Larger pieces of tissue were allowed to settle and supernatant was poured through gauze mesh into a tube containing FBS. Trypsinized cells were then pelleted and resuspended in appropriate media, then transferred into cell culture flasks and incubated in DMEM/F12-Hams (Sigma), each supplemented with 10% FBS (Gibco), 100U/ml penicillin, 100mg/ml streptomycin in a humidified incubator.

During the establishment of the tree shrew kidney-derived cell (TS-K5) cultures, non-adherent cells were lost during refreshing the medium. Only cells that attached to the culture flask were maintained and propagated by passage.

### Genome Analysis

The tree shrew genome, available in the NCBI database (BioProject Accession: PRJNA13971, 2* coverage, 2006) was analyzed by BLAST using known human or mouse IFN-λs, IFNλR1, IL10R2, OAS1 and Mx1 sequences, and the homologous contigs were retrieved in order to predict the IFN-λs transcripts. The transcripts of the retrieved contigs and scaffolds were predicted using GenScan (http://genes.mit.edu/GENSCAN.html) and in some cases were manually edited. Putative protein sequences for tree shrew IFN-λs, IFNλR1, and IL10R2 were compared with sequences in GenBank using BLASTP.

### Inducing IFN-λs Expression in TS-K5 Cells using Polyinosinic-Polycytidylic acid (poly I:C)

TS-k5 was seeded at 1*10^6^ cells/well in 6-well tissue culture plates and were transfected with 10 µg/ml polyinosinic-polycytidylic acid (poly I:C, Invivogen) using 10 µl Lipofectamine 2000 (Invitrogen), following the manufacturer’s protocols, and then incubated in a humidified incubator. Cells were harvested in lysis buffer (Fermentas) at 0, 1.5, 3, 6, 9, 12, 15 and 24h after transfection and stored at -80°C until the RNA was extracted according the manufacturer’s instructions.

### PCR Amplification of tsIFN-λ3 and its Receptor Subunits Genes and Quantitative Reverse Transcription PCR

Primers were synthesized based on the predicted sequences (Table 2), in order to amplify the full length coding region of the IFN-λ genes and the receptor genes by PCR. Total RNA extracted from TS-K5 cells, 3h after transfection with poly I:C, was used as a template for the amplification of IFN-λs genes. The first-strand cDNA was synthesized using oligo (dT)_18_ primer (RevertAid™ First Strand cDNA Synthesis Kit, Fermentas). The cDNA samples were diluted with TE buffer and used for PCR. Expression of the housekeeping gene, GAPDH, was measured for PCR and used as an internal control to allow equal amounts of template to be used for detecting IFN-λs expression. Briefly, each 50 µl PCR reaction contained 125 µM of each dNTP, 0.2 µM of each primer, 12 IU DreamTaq polymerase (Fermentas), and 2 µl cDNA template. The PCR program proceeded as follows: 1 cycle of 95°C for 3 min, 35 cycles of 95°C for 30s, 59°C-70°C for 30s, and then 72°C for 2 min, followed by a cycle of 72°C for 15 min.

**Table 2:** Primers used in this study.

Genes	primer	Sequence 5′-3′	Application
IFN-λ	IFN-λ-1F	TCGCGGACTCCCAGGCCGTACTGAGCAG	Part-length amplification
	IFN-λ-1R	TCAGACACAAGGTCCGGAGTGTGCCACCAT	Part-length amplification
	IFN-λ-2F	AGTTCCTGTCCCCAGGCCGT	Part-length amplification
	IFN-λ-2R	CCCTAGGGGACGAGTCAGCCA	Part-length amplification
	IFN-λ-3F	GCTGCAGGCTGGTGCTGA	Part-length amplification
	IFN-λ-3R	ACACAGGTCCCCACTGGC	Part-length amplification
	IFN-λ-4F	GAGTGTCCTGCGGCCTTGGAGACTGAGC	Part-length amplification
	IFN-λ-4R	ACACAGGTCCCCACTGGCGACACATC	Part-length amplification
IFN-λ3	IFN-λ3-1F	ATGGCCGGGGGCTGCAGGCTGGT	Full-length amplification
	IFN-λ3-1R	CTAGACACACAGGTCCCCACTGGCGACACA	Full-length amplification
	IFN-λ3-2F	GTGCTACGCCACATCCACTC	qRT-PCR
	IFN-λ3-2R	AGACTCCTTCTTCTCAGCCTCCT	qRT-PCR
IFNλR1	IFNλR1-1F	ATGTGGCGGGCCGGCCTGTGG	Full-length amplification
	IFNλR1-1R	CTAACCCTCATCCTCGTCCTCCCCTTCCTC	Full-length amplification
	IFNλR1-2F	TCACTCCTGCTCCTGCTGTG	qRT-PCR
	IFNλR1-2R	CATCTGTGCCCGCTGAAA	qRT-PCR
IL10R2	IL10R2-1F	CGTTGGGAATGGTGCCACCTCCTAAAAATG	Part-length amplification
	IL10R2-2F	TTAGTTTCATGAATTTGGGTGTTCTGATG	Full-length amplification
	IL10R2-R	CTAGGCGGATGTCAGCAGCAGGGGGTCG	Full-length amplification
	IL10R2-3F	ACTTCTGCTCTTCCCACTCTCTG	qRT-PCR
	IL10R2-3R	CGTTTGTTGCTCTCTGAGTCTTCT	qRT-PCR
OAS1	OAS-F	AACGTGTGGTGCACTGGTACTT	qRT-PCR
	OAS-R	CTTGGGCTTGGCTTTCTTCTT	qRT-PCR
Mx1	Mx1-F	GTGGTAGTCCCCTCGAATGTG	qRT-PCR
	Mx1-R	GGAGGAAACTGAAATAGGGGTGT	qRT-PCR
GAPDH	GAPDH-F	GGTCGGAGTAAACGGATTTGG	qRT-PCR
	GAPDH-R	AATGAAGGGGTCGTTGATGG	qRT-PCR

The amplified PCR products were extracted from agarose gels using a TIANgel mini purification kit (Tiangen). Purified PCR fragments were then cloned into pMD-19-T vector using pMD-19T vector (TaKaRa, D102A) for sequencing. M13 primers were used for sequencing (BigDye Terminator Cycle Sequencing Kit v3.1, Applied Biosystems with nonisotopic dye terminators in 20 µl reactions, according to the manufacturer’s protocols, and then analyzed on an Applied Biosystems 3130 XL Genetic Analyzer. Sequences were assembled manually using Seqman PRO (Lasergene) and Clone Manager 9.0 (Sci-Ed Software), and compared with sequences in GenBank using BLAST.

Next we performed qRT-PCR on total RNA extracted from cells. In brief, total RNA was extracted as described for PCR and qRT-PCR. The qRT-PCR primers were designed using Primer Express 3.0 (Applied Biosystems) with default parameter setting (Table 2). Reactions were carried out using LightCycler 480 SYBR Green I Master (Roche). For each reaction, 2 µl cDNA and a final concentration of 100nmol/L of each primer was used. The cycling profile consisted of an initial denaturation at 95°C for 5min followed by 45 cycles of 95°C for 10s, 55°C for 10s, followed by melt curve analysis. Both of the expression levels of the target genes (tsIFN-λ3, tsIFNλR1 and tsIL10R2) and their fold induction, as compared with the mock (tsOAS1 and tsMx1), were normalized relative to the housekeeping gene GAPDH.

### Sequence Analysis

The predicted tsIFN-λ3, tsIFNλR1, and tsIL10R2 sequences were deposited in the GenBank database as third-party annotated sequences. To determine the intron-exon organization of IFN-λs and their receptor genes, the full-length coding sequences were aligned with the corresponding sequences in the genome. Intron-exon maps of these genes were drawn using Fancy Gene 1.4 (http://host13.bioinfo3.ifom-ieo-campus.it/fancygene/). The protein sequences were deduced from the nucleic acid sequence using Primer 5.0 (PREMIER Biosoft International). Signal peptides were identified by SignalP 4.0 (http://www.cbs.dtu.dk/services/SignalP/). Multiple alignments were generated using Clustal-Omega (http://www.ebi.ac.uk/Tools/similarityandanalysis.html).

### Phylogenetic Analysis

Based on the amino acid alignments, we constructed phylogenetic trees using the Neighbor-joining method, maximum parsimony, and minimum evolution via MEGA5 with 1000 bootstrap replicates (http://megasoftware.net/mega.html) [54], [55]. The GenBank accession numbers for sequences used in the phylogenetic analysis are as follows: NM_172138-40, NM_000628, NM_170743, human; NM_001024673, NM_177396, NM_174851, NM_008349, mouse; NM_001107111, NM_001191868, rat; FJ581033-36, FJ581040, FJ581037, frog; XP_001501239, horse; GQ996936, FJ853390, pig; HQ201955-56, JN000223, JN000224, bat; NP_001108325, XP_855366, XM_535581, XM_850017, wolf; XP_001368442, opossum; EF587763, NM_204857, XM_417841, chicken; XP_001517931, platypus; NM_001076975, NM_001191497, cattle; XM_003307879, XM_531433, chimpanzee.

### ISGs Production in Response to tsIFN-λ3

The activity of the tsIFN-λ3 protein was determined by its ability to induce the production of ISGs. The ISG response was then determined using OAS1 and Mx1, both of which are known to be induced by type I IFNs in mammals [56], [57]. Supernatant was harvested at 3h and 12h after transfection and was used to treat the TS-K5 cells. Supernatant from Lipofectamine™ 2000 (Invitrogen) treated TS-K5 cells was used to as a mock control. Cells were incubated at 37°C for 6h and then harvested in lysis buffer (Fermentas) for extraction of total RNA. TsOAS1 and tsMx1 expression were determined by qRT-PCR.

### Protein Modeling

A three dimensional structures of the protein from its amino acid sequence were generated by Modeller. Easy Modeler, based on PerlTK, uses a very simple and easy interface to implement the features of Modeller 9.7 [58]. The extracellular structure of tsIFNλR1, tsIL10R2, human IFNλR1 and human IL10R2 were modeled using Easy Modeller 2.0 and Swiss PDB viewer with 3V2W [59]. The initial model building and structural alignment was performed and the modeled protein was visualized using PyMol 1.5 [60].
